# Supplementary material for: neuromaps: structural and functional interpretation of brain maps
Source: Nat Methods. 2022 Oct 6;19(11):1472–9. doi: 10.1038/s41592-022-01625-w (PMC9636018; doi:10.1038/s41592-022-01625-w)
Supplement: Supplementary file 1 — Supplementary Tables 1 and 2. [file 41592_2022_1625_MOESM1_ESM.pdf]

nature methods

Article

https://doi.org/10.1038/s41592-022-01625-w

neuromaps: structural and functional interpretation of brain maps

In the format provided by the authors and unedited

Table 1. **Published brain maps available in** `neuromaps`

Map name	*Coordinate system*	*Density*	*Citation*
Myelin map	fsLR	32k	Glasser et al. [30]
Cortical thickness	fsLR	32k	Glasser et al. [30]
Allen Human Brain Atlas PC1	fsaverage	10k	Markello et al. [28]
Delta power (2–4 Hz)	fsLR	4k	Van Essen et al. [29]
Theta power (5–7 Hz)	fsLR	4k	Van Essen et al. [29]
Alpha power (8–12 Hz)	fsLR	4k	Van Essen et al. [29]
Beta power (15–29 Hz)	fsLR	4k	Van Essen et al. [29]
Low gamma power (30–59 Hz)	fsLR	4k	Van Essen et al. [29]
High gamma power (60–90 Hz)	fsLR	4k	Van Essen et al. [29]
Intrinsic timescale	fsLR	4k	Van Essen et al. [29]
Developmental expansion	fsLR	164k	Hill et al. [19]
Evolutionary expansion	fsLR	164k	Hill et al. [19]
Functional gradient	fsLR	32k	Margulies et al. [32]
Inter-subject functional variability	fsaverage	1k	Mueller et al. [33]
NeuroSynth PC1	MNI152	2mm	Yarkoni et al. [34]
Cerebral blood flow (CBF)	fsLR	164k	Vaishnavi et al. [10]
Cerebral blood volume (CBV)	fsLR	164k	Vaishnavi et al. [10]
Oxygen metabolism (CMRO2)	fsLR	164k	Vaishnavi et al. [10]
Glucose metabolism (GMRGlu)	fsLR	164k	Vaishnavi et al. [10]
Allometric scaling (PNC)	CIVET	41k	Reardon et al. [22]
Allometric scaling (NIH)	CIVET	41k	Reardon et al. [22]
Neurotransmitter receptors/transporters	MNI152	—	Hansen et al. [14]

Table 2. **Standard coordinate system files available in** `neuromaps`

Coordinate system	*Density / resolution*	*Provided files*
fsLR	4k	midthickness, inflated, sphere
fsLR	8k	midthickness, inflated, sphere
fsLR	32k	midthickness, inflated, very inflated, sphere
fsLR	164k	midthickness, inflated, very inflated, sphere
fsaverage	3k	white, pial, inflated, sphere
fsaverage	10k	white, pial, inflated, sphere
fsaverage	41k	white, pial, inflated, sphere
fsaverage	164k	white, pial, inflated, sphere
CIVET	41k	white, midthickness, inflated, very inflated, sphere
MNI152	1mm	T1w, T2w, PD, brainmask
MNI152	2mm	T1w, T2w, PD, brainmask
MNI152	3mm	T1w, T2w, PD, brainmask
