# Supplementary material for: neuromaps: structural and functional interpretation of brain maps
Source: Nat Methods. 2022 Oct 6;19(11):1472–9. doi: 10.1038/s41592-022-01625-w (PMC9636018; doi:10.1038/s41592-022-01625-w)
Supplement: Supplementary file 2 — Reporting Summary [file 41592_2022_1625_MOESM2_ESM.pdf]

nature research

Corresponding author(s): Bratislav Misic

Last updated by author(s): Jun 14, 2022

Reporting Summary

Nature Research wishes to improve the reproducibility of the work that we publish. This form provides structure for consistency and transparency in reporting. For further information on Nature Research policies, see our Editorial Policies and the Editorial Policy Checklist.

Statistics

For all statistical analyses, confirm that the following items are present in the figure legend, table legend, main text, or Methods section.

n/a	Confirmed	
☒	☐	The exact sample size (*n*) for each experimental group/condition, given as a discrete number and unit of measurement
☒	☐	A statement on whether measurements were taken from distinct samples or whether the same sample was measured repeatedly
☐	☒	The statistical test(s) used AND whether they are one- or two-sided<br>*Only common tests should be described solely by name; describe more complex techniques in the Methods section.*
☒	☐	A description of all covariates tested
☐	☒	A description of any assumptions or corrections, such as tests of normality and adjustment for multiple comparisons
☐	☒	A full description of the statistical parameters including central tendency (e.g. means) or other basic estimates (e.g. regression coefficient) AND variation (e.g. standard deviation) or associated estimates of uncertainty (e.g. confidence intervals)
☐	☒	For null hypothesis testing, the test statistic (e.g. *F*, *t*, *r*) with confidence intervals, effect sizes, degrees of freedom and *P* value noted<br>*Give P values as exact values whenever suitable.*
☒	☐	For Bayesian analysis, information on the choice of priors and Markov chain Monte Carlo settings
☒	☐	For hierarchical and complex designs, identification of the appropriate level for tests and full reporting of outcomes
☐	☒	Estimates of effect sizes (e.g. Cohen's *d*, Pearson's *r*), indicating how they were calculated

Our web collection on statistics for biologists contains articles on many of the points above.

Software and code

Policy information about availability of computer code

Data collection	Data collection software includes the Connectome Workbench (v1.5.0, https://www.humanconnectome.org/software/connectome-workbench).
Data analysis	All code used for data processing, analysis, and figure generation directly relies on the following open-source Python packages: BrainSMASH (v0.11.0), BrainSpace (v0.1.2), IPython, Jupyter, Matplotlib (v3.5.0), NiBabel (v3.2.1), Nilearn (v0.8.1), NumPy (v1.21.5), Pandas (v1.3.3), PySurfer, Scikit-learn (v1.1.1), SciPy (v1.6.2), Seaborn (v0.11.2), and SurfPlot (v0.1.0; https://github.com/danjgale/surfplot). Additional software used in the reported analyses includes CIVET (v1.1.12 and v2.1.1, http://www.bic.mni.mcgill.ca/ServicesSoftware/CIVET), FreeSurfer (v6.0.0 and v5.3.0, http://surfer.nmr.mgh.harvard.edu/), and the Connectome Workbench (v1.5.0, https://www.humanconnectome.org/software/connectome-workbench, which is used in HCP's minimal preprocessing pipeline).

For manuscripts utilizing custom algorithms or software that are central to the research but not yet described in published literature, software must be made available to editors and reviewers. We strongly encourage code deposition in a community repository (e.g. GitHub). See the Nature Research guidelines for submitting code & software for further information.

Data

Policy information about availability of data

All manuscripts must include a data availability statement. This statement should provide the following information, where applicable:

- Accession codes, unique identifiers, or web links for publicly available datasets
- A list of figures that have associated raw data
- A description of any restrictions on data availability

Data used in the present analyses are publicly available on GitHub (https://github.com/netneurolab/neuromaps).The schizophrenia deformation-based

morphometry map used in Fig. 4 is derived from the NUSDAST dataset available at https://central.xnat.org/.The Human Connectome Project database is openly available at https://db.humanconnectome.org/data/projects/HCP_1200.

Field-specific reporting

Please select the one below that is the best fit for your research. If you are not sure, read the appropriate sections before making your selection.

☒ Life sciences ☐ Behavioural & social sciences ☐ Ecological, evolutionary & environmental sciences

For a reference copy of the document with all sections, see nature.com/documents/nr-reporting-summary-flat.pdf

Life sciences study design

All studies must disclose on these points even when the disclosure is negative.

Sample size	Transformations in the present software toolbox were generated on a cohort of 1045 HCP subjects. Sample size was not chosen, rather, all subjects in the HCP dataset were used.
Data exclusions	HCP subjects that did not successfully complete the CIVET processing pipeline were omitted.
Replication	The present manuscript proposes a software toolbox and is not experimental. Nonetheless, results are fully replicable using our open code and data. Analyses in Figure 5 were repeated across different coordinate systems.
Randomization	The present manuscript proposes a software toolbox and is not experimental. There were no experimental groups and therefore there was no randomization. Each brain map is averaged across all subjects in the sample.
Blinding	The present manuscript proposes a software toolbox and is not experimental. There were no experimenal groups and therefore there was no blinding. Each brain map is averaged across all subjects in the sample.

Reporting for specific materials, systems and methods

We require information from authors about some types of materials, experimental systems and methods used in many studies. Here, indicate whether each material, system or method listed is relevant to your study. If you are not sure if a list item applies to your research, read the appropriate section before selecting a response.

Materials & experimental systems

n/a	Involved in the study	
☒	☐	Antibodies
☒	☐	Eukaryotic cell lines
☒	☐	Palaeontology and archaeology
☒	☐	Animals and other organisms
☒	☐	Human research participants
☒	☐	Clinical data
☒	☐	Dual use research of concern

Methods

n/a	Involved in the study	
☒	☐	ChIP-seq
☒	☐	Flow cytometry
☐	☒	MRI-based neuroimaging

Magnetic resonance imaging

Experimental design

Design type	Structural MRI
Design specifications	No trials
Behavioral performance measures	No behavioural measures

Acquisition

Imaging type(s)	Structural MRI
Field strength	3T
Sequence & imaging parameters	Structural modalities were acquired on a Siemens Skyra 3T scanner and included a T1-weighted MPRAGE sequence at an isotropic resolution of 0.7mm, and a T2-weighted SPACE at an isotropic resolution of 0.7mm. More details on imaging protocols and procedures are available at http://protocols.humanconnectome.org/HCP/3T/imaging-

protocols.html.

Area of acquisition | Whole-brain

Diffusion MRI ☐ Used ☒ Not used

Preprocessing

Preprocessing software	Preprocessing was done using FSL 5.0.6, FreeSurfer 5.3.0-HCP, and Connectome Workbench v1.1.1.
Normalization	Image processing includes correcting for gradient distortion caused by non-linearities, correcting for bias field distortions, and registering the images to a standard reference space.
Normalization template	fs_LR_32k surface mesh
Noise and artifact removal	No artifact removal was preformed outside of the preprocessing and normalization.
Volume censoring	No volume censoring was performed.

Statistical modeling & inference

Model type and settings	No model was applied
Effect(s) tested	No effect was tested

Specify type of analysis: ☒ Whole brain ☐ ROI-based ☐ Both

Statistic type for inference (See Eklund et al. 2016)	NA
Correction	NA

Models & analysis

n/a	Involved in the study
☒	☐ Functional and/or effective connectivity
☒	☐ Graph analysis
☒	☐ Multivariate modeling or predictive analysis
